# Supplementary material for: Incorporation of a cost of deliberation time in perceptual decision making
Source: bioRxiv. 2024 Jan 31:2024.01.31.578067. Preprint. [Version 1] doi: 10.1101/2024.01.31.578067 (PMC10862799; doi:10.1101/2024.01.31.578067)
Supplement: Supplement 1 [file NIHPP2024.01.31.578067v1-supplement-1.pdf]

bioRxiv preprint doi: https://doi.org/10.1101/2024.01.31.578067; this version posted January 31, 2024. The copyright holder for this preprint (which was not certified by peer review) is the author/funder, who has granted bioRxiv a license to display the preprint in perpetuity. It is made available under aCC-BY 4.0 International license.

Supplementary information

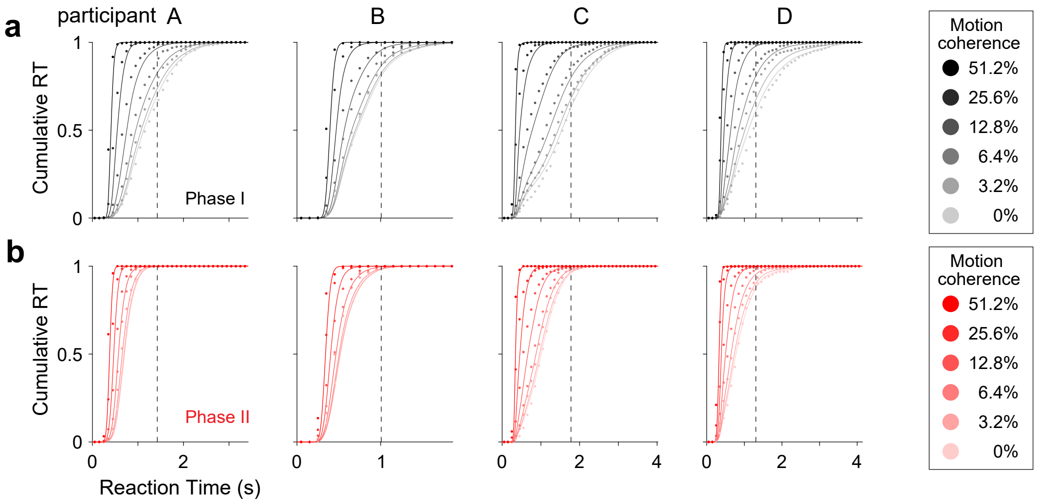

Figure S1. Reaction-time distribution across motion strengths predicted by *npb-DDM*
Cumulative distribution of observed RT (circles, 100 ms bins) and RT predicted by *npb-DDM* (curves) in Phase I (a) and II (b). Trials with a provisional deadline were excluded from the data in Phase II. Vertical dashed lines show mean provisional cancellation time for each participant, calculated from Phase II trials with provisional deadlines.

bioRxiv preprint doi: https://doi.org/10.1101/2024.01.31.578067; this version posted January 31, 2024. The copyright holder for this preprint (which was not certified by peer review) is the author/funder, who has granted bioRxiv a license to display the preprint in perpetuity. It is made available under aCC-BY 4.0 International license.

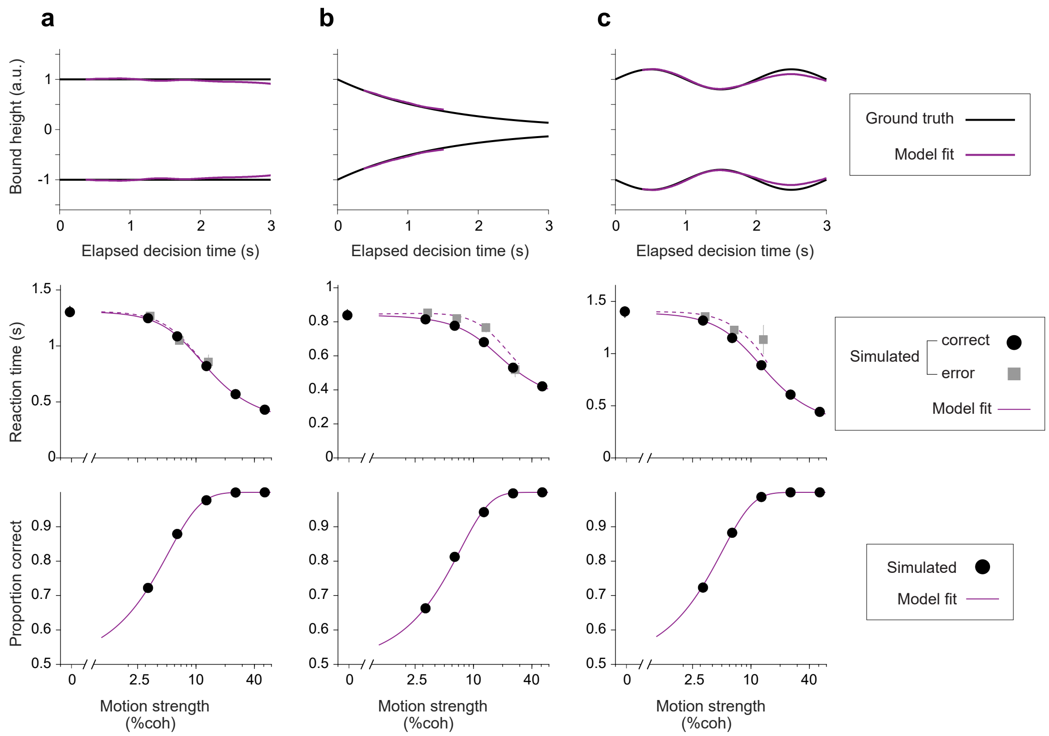

Figure S2. Validation of the *npb-DDM*

(a) We simulated 1,000 trials per motion coherence of a drift-diffusion model with the decision-termination bounds depicted by the black curves in the top panel. Mean reaction time (middle panel) and proportion correct (bottom panel) are shown as a function of motion strength (circles and squares). Red curves were obtained from fits of the *npb-DDM*.

(b-c) Same as panel (a) but for decision-termination bounds that decay exponentially (b) or oscillate (c). In all cases, the *npb-DDM* was able to recover the true shape of the bounds and fit the choice and RT data.

bioRxiv preprint doi: https://doi.org/10.1101/2024.01.31.578067; this version posted January 31, 2024. The copyright holder for this preprint (which was not certified by peer review) is the author/funder, who has granted bioRxiv a license to display the preprint in perpetuity. It is made available under aCC-BY 4.0 International license.

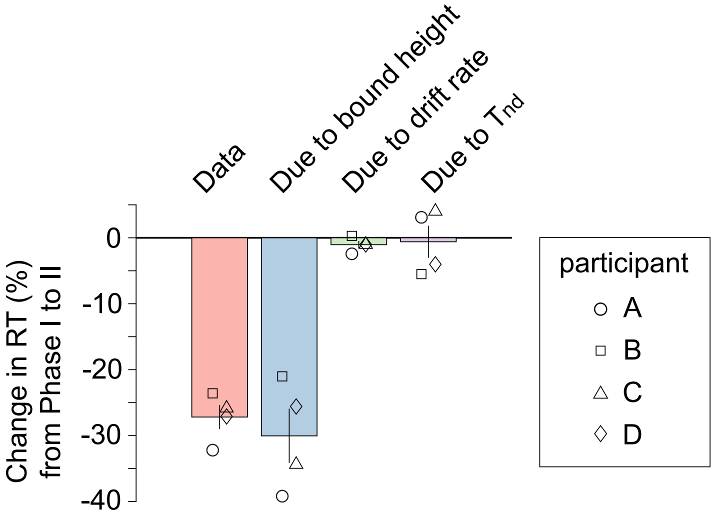

Figure S3. RT reduction due to the change in each model parameter from Phase I to II
Reduction in mean RT (%) from Phase I to II. Bars indicate mean across participants. Individual participant data are indicated by the open symbols. The leftmost (red) bar corresponds to the experimentally observed change in RT from Phase I to II. The next three bars correspond to model expectations. These are obtained from numerical solutions of the *npb-DDM* with parameters fit to the Phase I data, except for one of the parameters that is replaced with the value obtained from fitting Phase II data. The replaced parameters are bound height (blue bar), drift rate (green), and mean non-decision time (purple). The reductions in mean RT are significantly different from 0% for the changes due to bound height (p=0.0027; one-tailed t-test), but not for the changes due to drift rate (p = 0.078) or T_{nd} (p = 0.41).
